# O.4.3-11 The integration of Brain Gym Principle with Aerobic Dance for developing a new physical education instruction

**DOI:** 10.1093/eurpub/ckad133.197

**Published:** 2023-09-11

**Authors:** Rungrawee Samawathdana, Suthana Tingsabhat, Thanyarat Seenumkam, Rawisara Vathagavorakul, Waris Wongpipit

**Affiliations:** Chulalongkorn University, Thailand; rungrawee1113@gmail.com; Chulalongkorn University, Thailand; rungrawee1113@gmail.com; Chulalongkorn University, Thailand; rungrawee1113@gmail.com; Chulalongkorn University, Thailand; rungrawee1113@gmail.com; Chulalongkorn University, Thailand; rungrawee1113@gmail.com

## Abstract

**Purpose:**

The purpose of the study was to demonstrate a new Physical Education (PE) instruction by integrating the concept of Brain Gym (BY) Principles with Aerobic Dance (AD) to enhance cognitive and physical movement skills in secondary school students.

**Methods:**

The study was a systematic documentary research by reviewing BY and AD related research paper. The inclusion criteria for selecting documents were reliability, representativeness, and meaning. These findings were reviewed using five experts in Physical education, brain gym, and aerobics dance exercise. The Item Objective Congruence Index (IOC) was certified with IOC=1, as the basis for considering and rechecking the results of the study.

**Results:**

The new PE instruction composes of five steps: 1) introducing, reviewing, and checking the readiness of learning; 2) instructing, demonstrating, and rechecking; 3) practicing the brain and physical movement; 4) concluding and reflecting; and 5) cool down. Moreover, the learning activities should include four groups of the mix in low and high impact aerobics dance movements which were 1) Cross over; 2) Lengthening; 3) Energizing; and 4) Useful movement.

**Conclusions:**

The brain gym principles benefits learner in cognitive function, which enhancing in learning, communication ability, attention, memory, academic performance. While aerobic dance activity helps learners to improve the physical movement abilities, increases blood circulation, decreasing cholesterol levels, and enjoyment in learning. This research demonstrates the process of developing a new PE approach using documentary research qualifies by PE expertise and found that the new approach composed of five step and must be adapted brain gym and aerobic exercises principles. Hence, this new PE teaching approach have been implemented using quasi-experimental research and approved to publish in a qualifies publisher in Thailand.

